# Setting and Hardening Behaviour of Alkali-Activated Landfilled Fly Ash–Slag Binder at Room Temperature

**DOI:** 10.3390/ma13143130

**Published:** 2020-07-14

**Authors:** Wei Liu, Lin Lin, Shuping Wang, Xiaoqin Peng, Bobo Wu, Keke Sun, Lu Zeng

**Affiliations:** 1 Guangdong Provincial Key Laboratory of Durability for Marine Civil Engineering, Shenzhen University, Shenzhen 518060, China; liuwei@szu.edu.cn; 2 Shenzhen Durability Centre for Civil Engineering, Shenzhen University, Shenzhen 518060, China; 3 College of Materials Science and Engineering, Chongqing University, Chongqing 400045, China; lynn_is@126.com (L.L.); pxq01@cqu.edu.cn (X.P.); a842296893@163.com (B.W.); 20170901001@cqu.edu.cn (K.S.); zenglu@cqu.edu.cn (L.Z.); 4 Department of Project Management, Zunyi Transportation and Tourism Investment (Group) Co., Ltd, Zunyi 563000, China

**Keywords:** landfilled fly ash, alkali activation, setting time, strength development, microstructure

## Abstract

A geopolymer is normally considered an environmentally friendly binder due to the utilisation of industrial wastes. This study focusses on the potential of geopolymer preparation at room temperature from landfilled fly ash (LFA) which has been discharged to the land for more than three years. To accelerate the reaction process, 20–30 wt.% LFA was replaced by ground-granulated blast-furnace slag (GGBS). The effect of water glass modulus, Na_2_O content, water-to-solid ratio, and GGBS content on the setting time and strength development of the binder was discussed. Results showed that to activate LFA, the optimal value of the sodium silicate modulus for alkaline solution was 1.4–1.6 with a Na_2_O content of 10%, and the water-to-solid ratio was 0.4. In addition, the setting time of the binder reduced with increasing content of GGBS replacement, and the compressive strength increased due to the coexistence of C–(A)–S–H and zeolite-like phases. The maximum compressive strength of the binder was 29.2 MPa after 56 days of curing. The relatively low strength was likely due to the absence of the Q^4^ unit with a three-dimensional structure.

## 1. Introduction

Ordinary Portland cement (OPC) and concrete are considered non-sustainable construction materials due to the high emission of greenhouse gas and large amount of energy consumption during their manufacturing process [1]. It has been reported that industrial cement contributes 7% of greenhouse gas emissions and consumes approximately 10% of energy worldwide, annually [2]. Some researchers have even suggested that the only way to halt global warming is to reduce the emission of carbon dioxide caused by human activities to zero, and cement production was included [3]. One proposed solution is to replace Portland cement in concrete by industrial wastes, such as fly ash.

Fly ash is a by-product of coal combustion, mainly produced at power stations. It has fine particles with a size smaller than 75 μm, as it is collected from flue gases by electrostatic precipitators [4]. The annual production of coal fly ash is approximately 1400 million tons worldwide [5], which includes around 600 million tons in China with the utilisation rate estimated to be 75% [6]. Currently, fly ash is predominantly used in the construction industry, for example, as a supplementary cementitious material to replace cement in concrete in China. The glassy structure with reactive SiO_2_ and Al_2_O_3_ components makes it possible to react with Ca(OH)_2_, a hydration product of cement. On the other hand, 25% of the fly ash which is not utilised is still disposed of in landfill. The cumulative emission was approximately 2.5 billion tons in 2017, and it was estimated that 150 million tons are disposed on the land each year in China [7]. This creates serious environmental problems. Meanwhile, long-term weathering causes transformation in the physical and chemical composition [8,9]. Reactive components leach out and the hydraulic potential of landfilled fly ash (LFA) is much lower than that of conventional fly ash, which is usually not suitable to be used in the cement and concrete industry [10,11]. Therefore, more effective technology is demanded to reduce the landfilling as well as to reduce the greenhouse gases [9].

Alkali-activated fly ash (fly ash-based geopolymer) is considered as an environmentally favourable alternative to Portland cement for the lower amount of CO_2_ emission, massive waste recycling and lower cost [12,13,14,15,16]. Furthermore, a geopolymer has advantages of high strength, and excellent resistance to fire, acid and sulphate attack [13,17,18], which makes it attractive in this application. A case study conducted by Sandanayake et al. [19] showed that a fly ash-based geopolymer gave a reduction of 3.63–41.57% for the CO_2_ emission, and 23.80–30.25% reduction for production cost. However, some researchers argue that fly ash-based materials do not provide optimal results of CO_2_ emissions for geopolymers as a viable alternative to cement concrete, not only because of the high amount of alkali solution used, but also the requirement of heat curing to accelerate the reaction [20,21,22,23]. For the geopolymer prepared from Class F fly ash in particular, its structure development at ambient temperature was slow due to the high content of chemically inactive glassy phases in the source material [24].

Interestingly, it was found that the use of solid materials containing CaO can accelerate the setting of Class F fly ash-based geopolymer and enhance its strength under ambient conditions. Portland cement was used as an accelerator agent to improve the early strength and final strength of fly ash-based geopolymer [21,25]. The authors suggested the optimal replacement of fly ash by Portland cement to prepare a geopolymer at room temperature was 10–15%, and Portland cement content at this range could reduce microcracks in geopolymer concrete. In addition, it was also reported that partial replacement of fly ash by OPC can reduce the amount of alkali solution in the geopolymer [5], which makes the geopolymer more sustainable. Other investigations have focused on the influence of ground-granulated blast-furnace slag (GGBS) on the properties of fly ash-based geopolymers at room temperature. Results indicated that the compressive strength of the geopolymer was optimised by increased amounts of GGBS, and the structure became more compacted due to the formation of C–S–H gel (where C = CaO, S = SiO_2_ and H = H_2_O) [26,27,28,29,30,31]. It is believed that the co-existence of C–S–H gel and a zeolite-like phase in geopolymer products can improve the mechanical properties and durability of the geopolymer [32].

In the review above, it is found that few investigations have been done on the geopolymer system of landfilled fly ash. The setting and hardening behaviour of LFA-based geopolymer is still unclear. Meanwhile, the geopolymeric process of LFA-based geopolymer, which is highly dependent on the compositions of the material, will be different from the conventional fly ash. This study aims to investigate the potential of preparation of a sustainable LFA-based geopolymer binder at room temperature. The effect of alkali activator concentration and GGBS content on the setting time and compressive strength of the binder is discussed based on the reaction process and microstructure development.

## 2. Materials and Methods

### 2.1. Raw Materials

**Figure 1:**
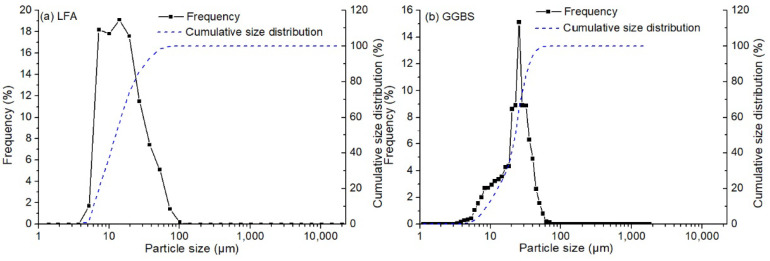
Particle size distribution of (**a**) landfilled fly ash (LFA), and (**b**) GGBS.

**Figure 2:**
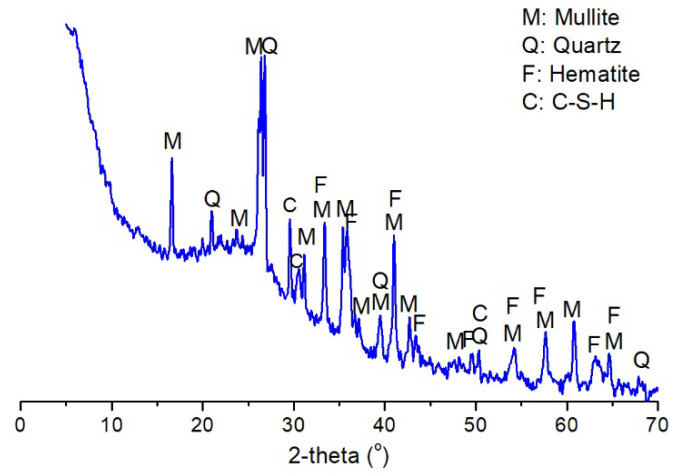
X-ray diffraction (XRD) patterns of LFA.

LFA was supplied by the Yuanfeng Company (Chongqing, China). The fly ash had been discharged to the land over a period of three years to thirty years. The caking phenomenon was observed in the material. It had a high impurity content attributed to the growth of plants. The moisture content of the material was higher than 17%. Before being used, the fly ash was oven dried at 105 °C for 24 h until the moisture was removed. Then, coarse particles larger than 0.63 mm were removed by sieving. GGBS was supplied by the Dixiang building materials company (Chongqing, China). The chemical compositions of these raw materials are performed according to the methods for chemical analysis of cement suggested in Chinese Standard (GB/T 176-2017), and results are shown in Table 1. The ignition loss of the oven-dried LFA was up to 10.56%, higher than the required value in GB/T 1596-2017. The particle size distributions of LFA and GGBS are shown in Figure 1. The D50 size (the size at the cumulative amount of 50% in volume) of LFA was 12.5 μm, and it was 23.0 μm for GGBS. Their specific surface areas were 344 m^2^/kg and 410 m^2^/kg, respectively. X-ray diffraction (XRD) results showed that its mineral composition included mullite, α-quartz, hematite ,and calcium silicate hydrate based on the powder diffraction files (PDF), corresponding to the file numbers of PDF 79-1276, PDF 82-0511, and PDF 89-8103, respectively (Figure 2). Consequently, the pozzolanic activity index of the material at 28 d was only 0.623, based on the method described in GB/T 12957-2005.

The alkali solution used for activating the hybrid binder was a sodium silicate solution (generally called water glass) with moduli (*n*, SiO_2_/Na_2_O molar ratio) of 1.2, 1.4 and 1.6, respectively. 18.33 g, 13.75 g and 10.3 g sodium hydroxide (NaOH) tablets (supplied by Kelong Chemical Reagent Factory, Chongqing, China) were dissolved directly into 100 g commercial water glass with modulus of 2.74 (supplied by Chongqing Jinkou Chemical plant, Chongqing, China, 10.7% Na_2_O, 28.93% SiO_2_ and 49.87% H_2_O), respectively. The amount of water, which should be added in the solution, was determined based on the Na_2_O equivalent content (mass ratio of Na_2_O to solid materials) and water-to-solid mass ratio (w/s) of the paste.

### 2.2. Sample Preparation

The alkali-activated LFA-GGBS hybrid binders were prepared in a step by step procedure. LFA was replaced by GGBS with the weight ratios of between 20% and 30%. Initially, LFA was mixed with GGBS homogeneously. Alkali solution was then added in the solid mixture and mixed for approximately three minutes to make a homogenous paste. The paste was cast in a 40 × 40 × 40 mm^3^ cubic mould and cured in a standard room (>95% RH, 20 ± 2 °C) for 24 h before the specimen was removed. After removal from the mould, the specimen was still cured under standard conditions until the age for the measurement of compressive strength was achieved. In this study, the effect of the GGBS content of the solid materials, the modulus of the alkali solution, Na_2_O content and water-to-solid ratio on the properties of the hybrid binders were investigated. The parameters are listed in Table 2.

### 2.3. Characterisation of Samples

Setting time of the paste, i.e., the time in which the material loses its plasticity and becomes hardened, was measured by a standard Vicat equipment according to ASTMC 191-04. The initial setting time was determined when the penetration depth of the initial setting needle was 25 mm, and final setting time was decided when the needle was not able to penetrate in the paste and no obvious marks were observed on the surface of the paste. Compressive strength of the hardened paste was measured after curing for 3 days, 7 days, 28 days and 56 days (also expressed as 3 d, 7 d, 28 d and 56 d, respectively) under standard conditions, by a hydraulic universal testing machine following the Standard GB/T 17671-1999. The fluidity was tested according to GB/T 8077-2000. A mini-slump cone was used in the measurement. The paste was filled in the cone within 30 s from the completion of mixing. Then the flow ring was lifted and the timer was started simultaneously. The paste was allowed to spread for 30 s before the diameter of the spread in two directions was measured. The fluidity was the average flow diameter of the paste.

Temperature evolution, which can reflect the geopolymeric process of the hybrid binder, was evaluated based on the method described by Bai el al. [34]. The fresh paste was placed in a 200 mL cylinder thermos container and a thermocouple was embedded in the centre of the sample. Temperature was recorded at one-minute intervals immediately after casting, and the test lasted for approximately 18 h. The difference between the tested temperature for the mixture and the surrounding temperature was defined as the “temperature increase”. The profile of “temperature increase rate” was the differential of the temperature increase-time profiles

An electrodeless resistivity meter (CCR-2) supplied by Bwin Technology Limited was used to detect the electrical resistivity of the hybrid binder. The paste was placed in a cylindrical mould and the outside wall of the mould was slightly beaten to make sure the air bubbles were removed from the paste. This process was completed in 10 min. Data were recorded at one-minute intervals immediately after the samples were fixed in the container. The measurement lasted for 3 days.

X-ray powder diffraction (XRD) was used to analyse the mineral phases of the LFA and hybrid binders at different curing ages by an X-ray diffractometer (Spectris Pte. Ltd, Singapore) with Cu-Kα radiation (λ = 1.5406 Å). The scanning range was from 5° to 70° 2θ, and the scanning rate was 2°/min with step of 0.02°. The functional groups of the hydration products of the hybrid binders were characterised by Fourier transform infrared spectroscopy (FTIR, Nicolet iS50, Thermo Fisher Scientific, Waltham, MA, USA). Scanning electronic microscopy (SEM, Tescan Vega II, Tescan Ltd., Brno, Czech) was used to investigate the morphology of the hybrid binders. The instrument was supplied by Tescan Ltd. (Brno, Czech) and was equipped with a secondary electron detector and EDS (energy-dispersive spectroscopy, INCA Energy 3500 X, Oxford, UK). The geopolymerisation reaction was stopped by immersing the samples in ethanol liquid for two weeks. Before measurement, the samples were dried by vacuum at 40 °C and followed by coating in gold with a thickness of ca. 20 nm, to improve the electronic conductivity.

## 3. Results and Discussion

### 3.1. Setting and Hardening of the Binder

#### 3.1.1. Influence of Alkali Solution

**Figure 3:**
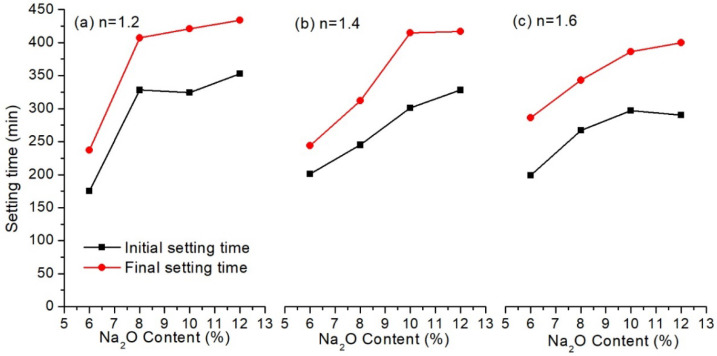
Setting time of alkali activated LFA-GGBS hybrid binder with different alkali solutions (GGBS content = 25%, water/solid ratio = 0.4): (**a**) modulus of water glass n = 1.2; (**b**) modulus of water glass n = 1.4; (**c**) modulus of water glass n = 1.6.

**Figure 4:**
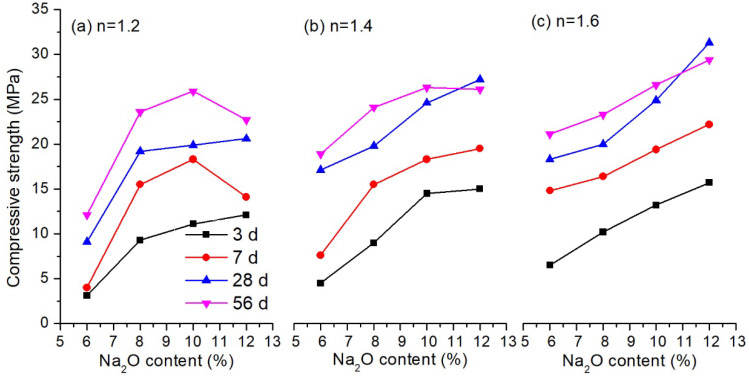
Compressive strength of alkali activated LFA-GGBS hybrid binder with different alkali solution (GGBS content = 25%, water/solid ratio = 0.4): (**a**) modulus of water glass n = 1.2; (**b**) modulus of water glass n = 1.4; (**c**) modulus of water glass n = 1.6.

In this section, the influence of Na_2_O content and modulus of alkali activator on the setting time and compressive strength of the alkali activated LFA-GGBS hybrid binders were investigated. GGBS content of the solid materials was 25%, and the water-to-solid weight ratio was 0.4. Results are shown in Figure 3 and Figure 4, respectively.

Figure 3 shows that the initial setting time of the hybrid binders was between 180 min and 330 min, while the final setting time ranged from 240 min and 420 min. This agreed with the requirement of common Portland cement in GB 175-2007 that the initial setting time should be longer than 45 min, but the final setting time should be shorter than 600 min. It should be noted that the setting time increased dramatically with alkali content at all the moduli of the alkali solution applied in this study. This was probably because increasing Na_2_O content would have a negative effect on the dissolution of the CaO of the GGBS, which in turn results in a slower formation rate of C–S–H gel [14]. When Na_2_O content was kept constant, the setting of the hybrid binders was not significantly affected by the modulus of the alkali solution, as shown in the figure.

A different tendency was observed from the results of compressive strength shown in Figure 4. It can be seen that a higher Na_2_O content generally provided a higher compressive strength of the hybrid binders, despite the increased setting time. For example, when the modulus was 1.2, the compressive strength of the hybrid binder with 6% Na_2_O content was 3.1 MPa, 4.0 MPa, 9.1 MPa and 12.1 MPa for 3 d, 7 d, 28 d and 56 d, respectively. When the Na_2_O content increased to 12%, the compressive strength was 12.1 MPa, 14.1 MPa, 20.6 MPa and 22.7 MPa, corresponding to 3 d, 7 d, 28 d and 56 d curing, respectively. The reason is that the dissolution of the glassy-like structure of LFA and GGBS usually occurs in a strong alkali environment. The pH value of the alkali solution was increased by a higher Na_2_O content, which will promote the amount of dissolved fly ash and slag particles [35]. As a result, more gels are formed to produce a denser structure, and the compressive strength is improved. However, it should be noted that a high Na_2_O content possibly has a negative effect on the strength development. When the Na_2_O content was 12%, the 56-day strength of the hybrid binder was lower than the 28-day strength at the modulus of 1.4 and 1.6 (Figure 4b,c).

In addition, the compressive strength was also improved by increasing the modulus of the water glass. For the modulus of 1.6, the 3-day strength was 6.5 MPa, 10.2 MPa, 13.2 MPa and 15.7 MPa, while 56-day strength was 21.1 MPa, 23.3 MPa, 26.6 MPa and 29.4 MPa, corresponding to the binder with Na_2_O content of 6%, 8%, 10% and 12%, respectively. This showed that an alkali solution with a modulus of 1.6 was suitable to activate the LFA-GGBS hybrid binder. It is known from the experiment that 12% Na_2_O is neither beneficial to the setting of the binder, nor to the strength development at the later age. When the Na_2_O content was 10%, no great difference of the compressive strength was observed at the modulus between 1.4 and 1.6. For further study, therefore, an alkali solution with Na_2_O content of 10% and modulus of 1.4–1.6 can be used as an activator.

#### 3.1.2. Influence of Water-to-Solid Ratio

**Figure 5:**
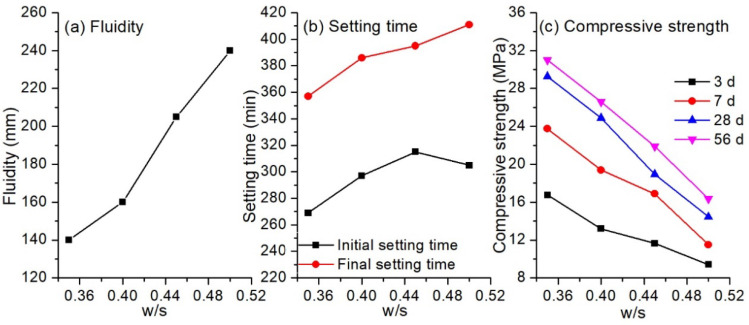
Setting and hardening properties of alkali-activated LFA-GGBS hybrid binder with different water/solid ratios (GGBS content = 25%, Na_2_O content = 10%, n = 1.6): (**a**) fluidity of the binder; (**b**) Setting time of the binder; (**c**) compressive strength of the binder curing for different ages.

The influence of water-to-solid ratio (w/s) on the fluidity, setting time and compressive strength of the hybrid binder is shown in Figure 5. There is no doubt that the fluidity of the paste increased almost linearly with the water-to-solid ratio. When w/s ratio was 0.35, the fluidity was 138 mm. The paste was quite sticky and not suitable for casting. As the ratio increased to 0.45, the viscosity of the paste decreased dramatically with the fluidity increasing to 206 mm, and bleeding was observed. Results showed that the hybrid binder with a water-to-solid ratio of 0.40 provided a paste with suitable fluidity (160 mm) for casting. Similar to the increasing tendency of fluidity, the setting time also increased as the w/s ratios increased. The initial setting time and final setting time was 269 min and 357 min, respectively, for the binder with w/s ratio of 0.35, and the values were up to 305 min and 411 min at a w/s of 0.50. A linear decrease in compressive strength with increasing w/s ratios was present for all the cured ages. Three-day strength was 16.8 MPa at a w/s of 0.35, but this decreased by 44% to 9.4 MPa, whereas the 28-day strength of the binder decreased from 29.3 MPa to 14.4 MPa. The decrease of compressive strength was mainly due to the increased porosity of the binder as the w/s ratio increased [36].

#### 3.1.3. Influence of Ground-Granulated Blast-Furnace Slag (GGBS) Content

**Figure 6:**
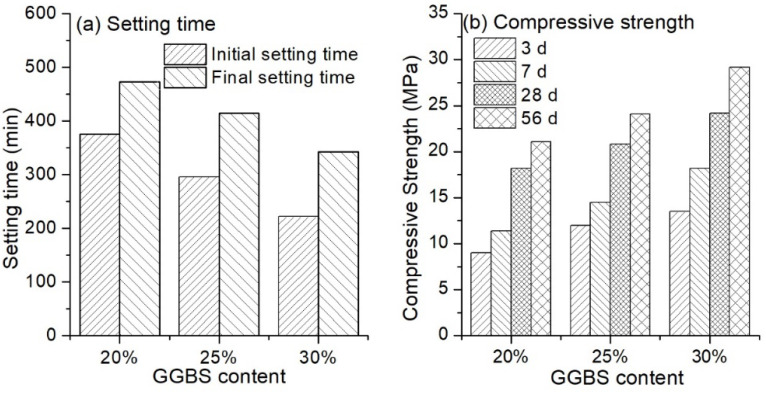
Setting time (**a**) and compressive strength (**b**) of alkali activated LFA-GGBS hybrid binder with different GGBS content (Na_2_O content 10%, n = 1.4, and w/s = 0.4).

Different GGBS content was used in the hybrid binder to investigate the variation of setting time and compressive strength, and results are shown in Figure 6. A remarkable decrease in the setting time was observed with an increase of GGBS content. The initial setting time was approximately 380 min for the binder containing 20%, and a decrease of around 80 min appeared with an increase of GGBS content of 5%. In terms of final setting time, the value was nearly 470 min for the hybrid binder with 20% GGBS, and it decreased to 343 min when the GGBS content was increased to 30%. The reason was that with the presence of a high amount of Ca ions (37.46% CaO content in GGBS), the polymerisation degree of GGBS was lower than that of LFA which contained 2.49% CaO [37]. In this case, GGBS was much easier to dissolve in the alkali solution to form hydration products, than fly ash. Consequently, the setting time was reduced. On the other hand, introducing more GGBS in the hybrid binder would improve the compressive strength, as shown in Figure 6b. When the GGBS content was 20%, the compressive strength was 9.0 MPa, 11.4 MPa, 18.2 MPa and 21.1 MPa for 3 d, 7 d, 28 d and 56 d curing, respectively. The values increased by 33.3%, 27.2%, 14.3% and 14.3%, respectively, at the GGBS content of 25%. For the binder with a GGBS content of 30%, the increase in compressive strength was 50.0%, 59.4%, 33.0% and 38.4% for 3 d, 7 d, 28 d and 56 d curing, respectively. This can likely be attributed to the increasing amount of C–S–H and C–A–S–H gels (where C = CaO, A = Al_2_O_3_, S = SiO_2_, H = H_2_O) with a higher content of GGBS [32]. Furthermore, with an increasing amount of GGBS replacement, the Si/Al ratio of the material increased, which would also improve the strength of the binder [35].

### 3.2. Reaction of the Hybrid Binder

#### 3.2.1. Isothermal Temperature Increase

**Figure 7:**
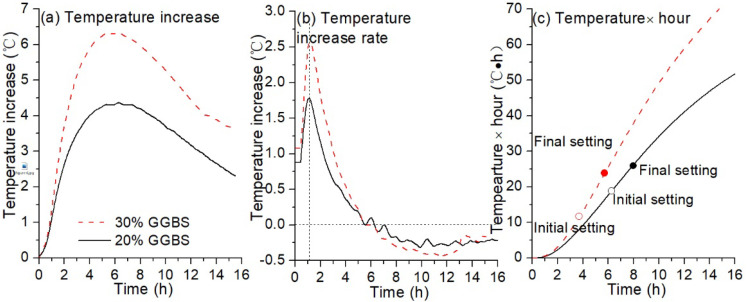
Temperature increase of alkali activated LFA-GGBS hybrid binder with different GGBS content (Na_2_O content 10%, n = 1.4, and w/s = 0.4): (**a**) temperature increase; (**b**) temperature increase rate; (**c**) temperature × hour.

Figure 7a–c show the temperature-increase with time plots for the alkali-activated hybrid binders with different GGBS contents for short-time curing. No obvious difference was observed between the temperature increase curves in the initial hour after the mixtures were prepared (Figure 7a,c). This is probably due to the dissolution of silicon and aluminium containing source materials in the high alkali solution [38]. After one-hour curing, the temperature increase became higher for the GGBS replacement of 30% than that for the 20%, and the rate of temperature-increase dramatically jumped up to a maximum value in approximately 10 min. This is likely ascribed to the faster dissolution rate of slag particles than fly ash particles [37], and in this case, a higher amount of geopolymerisation products will be produced at a higher replacement level of GGBS. In the following four to five hours, the rate of the temperature increase dramatically decreased to zero. This process was related to the fluidity loss of the paste, and setting occurred. It was found that the peak of temperature increase for the pastes of 30% and 20% GGBS content appeared at approximately 6 h and 7 h, respectively. The results somehow confirm the final setting time of the paste, as shown in Figure 6a. A decrease in the temperature increase rate followed and the rate of temperature increase became negative. The reason was that the experiment conducted in this study was under semi-adiabatic conditions and heat diffusion became dominant. It can be suggested that the geopolymerisation reaction slowed down at this period due to the formation of a more compacted structure. The maximum temperature increase provided by the binder containing 30% GGBS was 6.3 °C, approximately 46.5% higher than that provided by the binder with 20% GGBS. This indicates a higher total heat generated by the former hybrid binder, and consequently the binder showed a much higher compressive strength, as shown in Figure 6b.

#### 3.2.2. Electrical Resistivity

**Figure 8:**
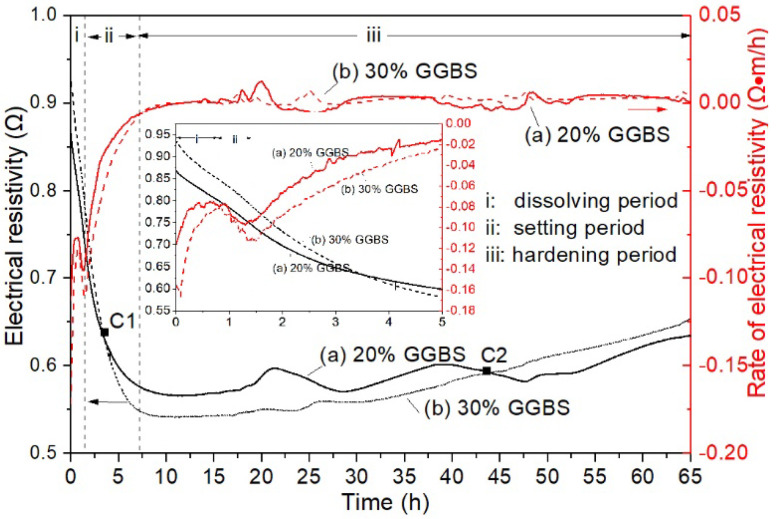
Electrical resistivity of alkali activated LFA-GGBS hybrid binder with different GGBS content (Na_2_O content 10%, n = 1.4, and w/s = 0.4): (a) 20% GGBS; (b) 30% GGBS content.

The electrical resistivity of the alkali activated hybrid binder is shown in Figure 8. Obviously, the variation of electrical resistivity was similar between the two hybrid binders with different GGBS content, such that three periods were included according to the rate of the electrical resistivity curves. However, the initial electrical resistivity was higher for the hybrid binder with 30% GGBS than the binder with 20%. This is probably ascribed to the higher specific surface area of GGBS than LFA, and the former paste was stickier than the latter. Electrical resistivity decreased dramatically as time increased, indicating the increased number of ions in the solution. This was a result of the dissolution of GGBS and LFA particles in the high pH solution. Thus, the rate of electrical resistivity became less negative corresponding to the period I, as shown in the figure. This process occurred in the first hour after the mixture was prepared. The following period (period II) was a setting period. In this period, GGBS and LFA particles are continuously dissolving. Meanwhile, polymerisation was possible as the ions concentration in the solution increased. As a result, the rate of electrical resistivity became much less negative, despite the fact that the electrical resistivity still decreased. In this case, the dissolution of the particles was also dominant. It should be noted that at the first cross point (C1), the value of the electrical resistivity of the binder with 30% GGBS content was lower than that of the binder with 20% GGBS. This point is related to the initial setting of the former binder, demonstrating that a higher amount of GGBS would accelerate the dissolution of the particles. At approximately seven hours after mixing, the electrical resistivity gradually increased, indicating the increased amount of polymerisation products. This was related to the hardening process of the binder [39]. The dissolution of the GGBS and LFA particles became difficult as the porosity decreased and the thickness of the polymerisation products on the particles increased. Consequently, polymerisation and diffusion of the ions became dominant. In addition, a second cross point (C2) was observed, and the electrical resistivity of the binder with 30% GGBS was higher than that of 20% GGBS at approximately 45 h after reaction. This is because GGBS shows higher reactivity with a lower degree of connectivity in its structure than fly ash [37]. In this case, the rate of dissolution and polymerisation of GGBS will be faster than fly ash. Therefore, the binder with 30% slag had shorter setting time and higher strength than those with lower GGBS content.

### 3.3. Microstructure of the Hybrid Binder

#### 3.3.1. X-ray Diffraction (XRD) Analysis

**Figure 9:**
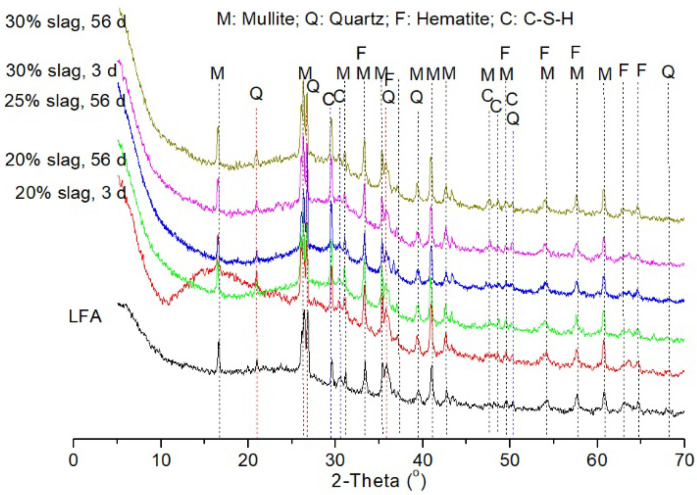
X-ray diffraction (XRD) patterns of alkali activated LFA-GGBS hybrid binder with different GGBS content at different curing ages.

Figure 9 gives the XRD patterns of the hybrid binder with different GGBS content at different curing ages. Compared with the pattern of LFA, humped shapes at 2θ of between 20° and 40° appeared in all the hybrid binders, indicating the formation of a C–S–H gel and a zeolite-like gel after alkali activation [40,41]. Despite that no obviously new peaks were created in the alkali-activated samples compared with LFA, the intensity of the peak with d-spacing of 0.302 nm (2θ of 29.2°) increased with an increased amount of GGBS content. This peak was characteristic for C–S–H gel [42], which is due to the dissolution and precipitation of the GGBS in the high alkaline solution. It demonstrated that the coexistence of C–S–H gel and zeolite-like gel could improve the mechanical properties of the hybrid binders as the compressive strength of the binder increased with GGBS content. Mineral phases such as mullite, quartz and hematite were not involved in the geopolymerisation reaction since these phases were still present in the hybrid binders, and no obvious difference was observed from the intensity of the XRD patterns.

#### 3.3.2. Fourier Transform Infrared (FTIR) Spectroscopy

**Figure 10:**
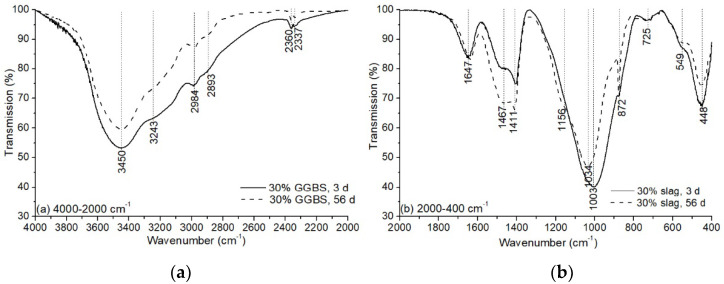
Fourier transform infrared (FTIR) spectrum of alkali-activated LFA-GGBS hybrid binders (Na_2_O content 10%, n = 1.6, and w/s = 0.4, 30% GGBS): (**a**) 4000–2000 cm^−1^; (**b**) 2000–400 cm^−1^.

FTIR analysis on the hybrid binder with 30% GGBS is shown in Figure 10. The bands appearing at the region of 2000–4000 cm^−1^ are attributed to the stretching vibration of hydrogen groups (Si–O–H). The vibration at 1647 cm^−1^ results from the bending of the hydroxyl band. These bands indicated the presence of weakly bound water molecules that were adsorbed on the surface on the geopolymeric products [41]. The decrease of intensity in the region of 2000–4000 cm^−1^ showed a reduced amount of water molecules as the reaction proceeded, demonstrating that the water molecules are consumed during the polycondensation reaction [43]. The bands located at the region of 1400–1500 cm^−1^ are the signal of asymmetric stretching vibration of ${\mathrm{CO}}_{3}^{2-}$, and the band position at 872 cm^−1^ is attributed to the bending vibration of the C–O bond of carbonates [43]. It was found that the amount of carbonation product was higher when the sample was cured for 56 days than curing for 3 days.

**Figure 11:**
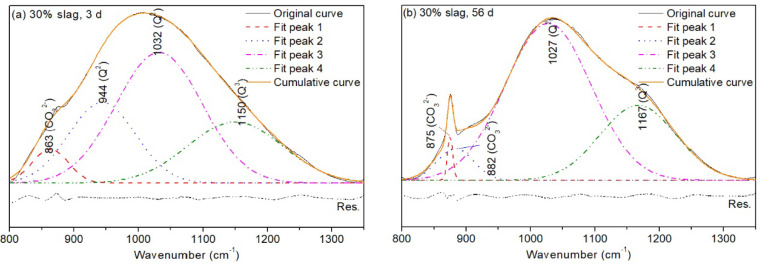
Deconvolution of Si–O band ranging from 800 to 1350 cm^−1^ of alkali activated LFA-GGBS hybrid binders with different GGBS content at different ages (Na_2_O content 10%, n = 1.6, and w/s = 0.4): (**a**) 30% GGBS, 3 d; (**b**) 30% GGBS, 56 d.

The bands positioned in the range of 800–1350 cm^−1^ are related to the stretching vibration of Si–O–R (with R = Si, Al, Na, etc.). The band at 1003 was ascribed to the Q^2^ units of Si–O–Si for 3-day curing (Figure 10b). The polymerisation degree of the binder increased for 56 days of curing as the vibration shifted to a higher wavenumber (1034 cm^−1^). In addition, a “shoulder” peak appeared at 1156 cm^−1^, representing the formation of Q^3^ units. For further understanding of the structural variation caused by curing age, the bands’ vibration between 800–1350 cm^−1^ were deconvoluted into four peaks. The results are shown in Table 3 and Figure 11.

In Figure 11, ${\mathrm{CO}}_{3}^{2-}$ vibration appeared at 863–882 cm^−1^ for different curing ages. A band with wavenumber of 944 cm^−1^ as a representative of Q^2^ units was present in the sample when cured for 3 days [44,45]. However, as the curing age increased to 56 days, this type of Q^2^ unit disappeared. Instead, the fitting area of a silica-rich gel with a vibration of 1027 cm^−1^ increased by 37.9%. This band is likely a representative of Q^2^ units with finger print structure [46], demonstrating the increased connectivity of the silicate chain by curing. Q^3^ units were present at 1150 cm^−1^ in the sample of 3-day curing. When the curing age increased to 56 days, Q^3^ units shifted to a higher vibration wavenumber (1167 cm^−1^) with an increased fitting area, indicating the higher degree of geopolymerisation of the structure. Q^4^ units were absent in the structure, indicating that alkali-alumina silicates with three-dimensional structure characteristics were not formed [29]. Consequently, although the compressive strength of the hybrid increased, the value was still not as high as expected.

#### 3.3.3. Scanning Electron Microscopy (SEM) Analysis

**Figure 12:**
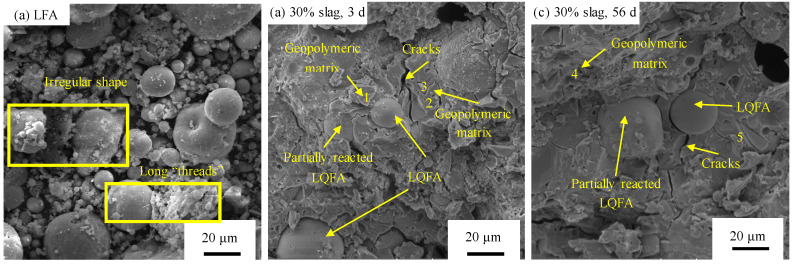
Scanning electron microscope (SEM) photographs of (**a**) LFA; (**b**) alkali-activated LFA-GGBS hybrid binders with 30% GGBS curing for 3 d; (**c**) alkali activate LFA-GGBS hybrid binders with 30% GGBS curing for 56 d.

The microscopy photographs of LFA and hybrid binder are shown in Figure 12. By contrast with the morphology of commercial fly ash, which consists of spherical and vitreous particles [41], particles with an irregular shape and rough surface were also found in LFA, and the size ranged from 1 μm to 40 μm, and coarse agglomerates with depositional bonding structure in the appearance of long “threads” can be observed in Figure 12a. This agrees with the description in [11]. It was caused by the weathering effect during the land filled period. Consequently, the reactivity decreased. Geopolymeric matrices were developed at three days of curing, despite the presence of unreactive fly ash particles (Figure 12b). In addition, micro-cracks with a width of approximately 2 μm were obvious in the geopolymeric matrix. The presence of cracks was probably caused by thermal stresses during the geopolymerisation process. When the curing time increased to 56 days, the size of cracks was reduced due to the reaction of fly ash such that more geopolymeric products were formed to fill the cracks. Energy-dispersive X-ray spectroscopy (EDX) measurements, shown in Table 4, indicate that the main geopolymeric products were the co-existence of N–A–S–H gel (where N = Na_2_O, A = Al_2_O3, S = SiO_2_ and H = H_2_O) and C–(A)–S–H gel. However, LFA was not completely consumed, even for 56-day of curing, as fly ash particles were still observed in the sample, which means the reactivity of LFA did not completely react at room temperature. Suitable curing will be required to accelerate the reaction for further investigation.

## 4. Conclusions

In this study, landfilled fly ash with low quality was activated by water glass to investigate the possibility of preparing a sustainable fly ash-based geopolymer at room temperature. Ground granular blast furnace slag was added to optimise its setting and hardening properties. Results showed that the hybrid binder was more reactive when the alkali content was 10% and the modulus of water glass was 1.4–1.6. The addition of GGBS was effective in accelerating the geopolymerisation of the hybrid binder to reduce the setting time and improve the strength. Compared to the binder with a GGBS content of 20% in the solid material, the initial setting time and final setting time of the binder with a GGBS content of 30% was reduced by 41.6% and 27.6%, respectively. In addition, the three-day strength was improved by 50.0%. This was due to the GGBS being more reactive than fly ash, and it can be dissolved more easily in the alkali solution to form N–A–S–H and C–(A)–S–H, based on the study of the isothermal temperature increase and electrical resistivity. Microstructural analysis also showed that the fly ash was still present in the binder, even for 56-day curing, with a compressive strength of 29.2 MPa, even though the geopolymerisation degree was greatly improved compared to that of 3 days of curing. Furthermore, Q^4^ units of alumina-silicates with a three-dimensional structure were not detected in the sample cured for 56 days. In this case, suitable curing should be adapted to accelerate the reaction of low-quality fly ash in a future study.

## Figures and Tables

**Table 1:** Chemical composition of landfilled fly ash (LFA) and ground-granulated blast-furnace slag (GGBS) (wt.%) [33].

Sample	SiO_2_	Fe_2_O_3_	Al_2_O_3_	CaO	MgO	Ignition Loss	Others
LFA	40.41	14.65	24.99	2.49	/	10.56	6.9
GGBS	33.75	1.36	13.55	37.76	7.65	/	5.93

**Table 2:** Parameters which influence the properties of alkali-activated LFA-GGBS hybrid binder.

Factors	Values
GGBS content	20%, 25% and 30%
Modulus of alkali solution (n)	n = 1.2, 1.4 and 1.6
Na_2_O content (mass ratio of solid materials)	6%, 8%, 10% and 12%
Water to solid ratio (w/s)	0.35, 0.40, 0.45 and 0.50

**Table 3:** Peak properties after deconvolution of alkali activated LFA-GGBS hybrid binders (Na_2_O content 10%, n = 1.6, and w/s = 0.4, 30% GGBS).

Band	3 d			56 d		
	Wavenumber (cm^−1^)	Area Fit (%)	FWHM	Wavenumber (cm^−1^)	Area Fit (%)	FWHM
${\mathrm{CO}}_{3}^{2-}$	863	5.17	66	875–882	6.76	10.1, 66.9
$Q^{2}$	944	22.85	122.5	/	/	/
$Q^{2}$ (Silica-rich gel)	1032	47.46	157	1027	65.46	157.5
$Q^{3}$	1150	24.52	172.9	1167	27.78	139.6

**Table 4:** The average content of different elements (%) for each point in Figure 12.

Elements	Ratio of the Element at Different Position				
	1	2	3	4	5
C	-	-	-	4.9	-
Na	3.55	8.95	3.26	16.9	3.75
Ca	20.00	4.35	12.50	21.63	7.87
O	50.42	67.96	60.15	49.45	64.52
Si	18.55	15.78	14.05	10.5	29.01
Al	7.48	2.95	5.76	-	4.57
Fe	6.19	-	-	-	-
Mg	-	-	4.28	-	-
